# Glutamatergic Resonance and Dopaminergic Stability: Intranasal Esketamine in Treatment-Resistant Depression with Comorbid ADHD and Autism Spectrum Disorder – A Case Report

**DOI:** 10.1192/j.eurpsy.2026.10430

**Published:** 2026-07-31

**Authors:** M. C. Angeletti, K. La Monica, V. Casati, G. Versaci, T. Prodi, F. Mazzoni, A. Guffanti, N. Brondino, V. Martiadis, R. Anniverno, B. M. Dell’Osso, M. Olivola

**Affiliations:** 1 Department of Mental Health and Addiction, ASST Fatebenefratelli Sacco; 2 Department of Biomedical and Clinical Sciences, University of Milan, Milan; 3 Department of Brain and Behavioural Sciences, University of Pavia; 4 Department of Mental Health and Addiction, ASST Pavia, pavia; 5 Department of Mental Health, ASL Napoli 1 Centro, Naples, Italy; 6 Department of Psychiatry and Behavioral Sciences, Bipolar Disorders Clinic, Stanford University, Stanford, United States; 7 ”Aldo Ravelli” Center for Nanotechnology and Neurostimulation; 8 Department of Psychiatry, University of Milan, Milan, Italy

## Abstract

**Introduction:**

Treatment-resistant depression (TRD) represents a major clinical challenge, particularly in the presence of neurodevelopmental comorbidities such as attention-deficit/hyperactivity disorder (ADHD) and autism spectrum disorder (ASD). Both conditions complicate illness trajectories and may limit the efficacy of conventional pharmacological strategies. Recent evidence suggests that intranasal esketamine, through modulation of glutamatergic neurotransmission and indirect stabilization of dopaminergic circuits, may constitute a novel therapeutic approach.

**Objectives:**

To describe the clinical effects, safety profile, and potential neurobiological implications of intranasal esketamine in a patient with TRD and comorbid ADHD and ASD.

**Methods:**

We report the case of a 24-year-old female with major depressive disorder unresponsive to multiple antidepressants and augmentation strategies, who presented with persistent depressive symptoms, severe functional impairment, and marked suicidality. She also met diagnostic criteria for ADHD and ASD. The patient was treated at the **Esketamine Outpatient Clinic of Macedonio Melloni Hospital (ASST Fatebenefratelli Sacco, Milan)**, where intranasal esketamine was administered according to approved TRD protocols, with close clinical monitoring. Outcome measures included standardized rating scales for depression, suicidality, and global functioning. Particular attention was devoted to the evolution of ADHD- and ASD-related symptomatology, with a focus on emotional regulation, attentional control, and social reciprocity.

**Results:**

A rapid reduction in depressive symptoms and suicidality was observed within the first weeks, with progressive functional recovery over subsequent sessions. Symptomatic improvement extended beyond mood, encompassing emotional regulation, attentional control, and social interaction, suggesting a broader modulatory effect on glutamatergic–dopaminergic networks. The treatment was well tolerated; transient dissociative symptoms occurred early but resolved spontaneously without discontinuation. No major adverse events were reported.

**Image 1:**
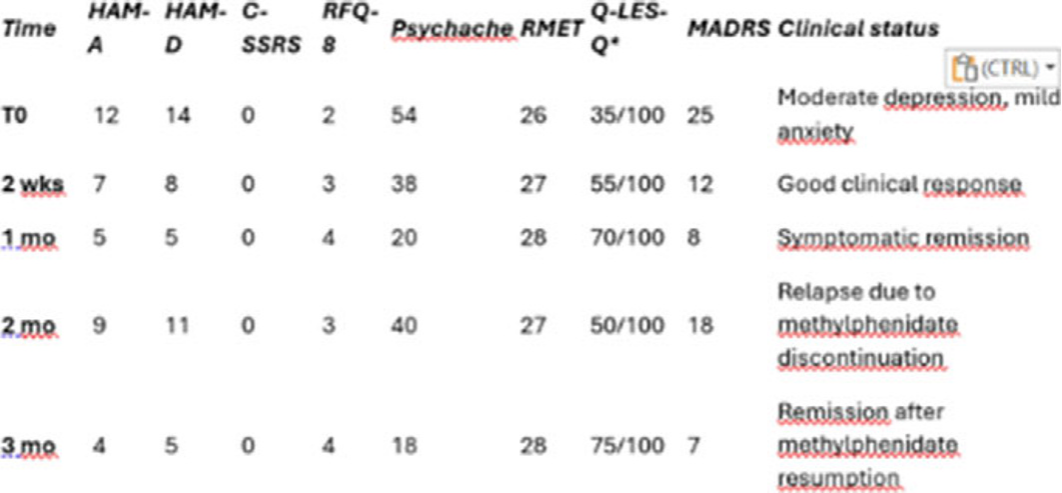
Image 1: Long description.

**Image 2:**
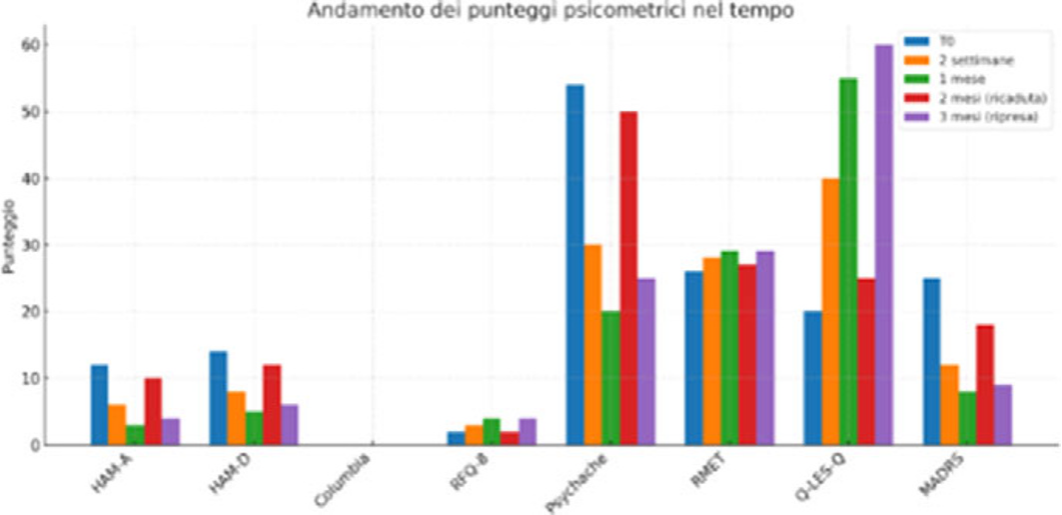
Image 2: Long description.

**Conclusions:**

This case highlights intranasal esketamine as a promising therapeutic option for TRD complicated by ADHD and ASD. Benefits extended beyond antidepressant response, encompassing attention and socio-emotional regulation, consistent with the hypothesis that esketamine may enhance neural integration through glutamatergic resonance and dopaminergic stabilization. Although based on a single observation, these findings underscore the potential role of esketamine in addressing the multidimensional burden of TRD with neurodevelopmental comorbidities and warrant further investigation in controlled studies.

**Disclosure of Interest:**

None Declared

## Long descriptions

### Image 1: Long description

The table consists of nine columns and five rows of data. The columns are labeled: Time, H A M dash A, H A M dash D, C dash S S R S, R F Q dash 8, Psychache, R M E T, Q dash L E S dash Q, M A D R S, and Clinical status.

* Row 1 (T 0): Scores are 12, 14, 0, 2, 54, 26, 35/100, 25. Clinical status: Moderate depression, mild anxiety.

* Row 2 (2 wks): Scores are 7, 8, 0, 3, 38, 27, 55/100, 12. Clinical status: Good clinical response.

* Row 3 (1 mo): Scores are 5, 5, 0, 4, 20, 28, 70/100, 8. Clinical status: Symptomatic remission.

* Row 4 (2 mo): Scores are 9, 11, 0, 3, 40, 27, 50/100, 18. Clinical status: Relapse due to methylphenidate discontinuation.

* Row 5 (3 mo): Scores are 4, 5, 0, 4, 18, 28, 75/100, 7. Clinical status: Remission after methylphenidate resumption.

Overall trends show an initial improvement in scores (lower H A M and M A D R S, higher Q dash L E S dash Q) followed by a temporary worsening at month 2 and recovery by month 3.

Navigate back to Image 1:.

### Image 2: Long description

The y-axis is labeled Punteggio (Score) ranging from 0 to 60. The x-axis lists eight psychometric tests. A legend in the top right identifies five time points: T 0 (blue), 2 settimane (orange), 1 mese (green), 2 mesi ricaduta (red), and 3 mesi ripresa (purple).

* H A M dash A: Scores decrease from T 0 to 1 month, peak again at 2 months, and drop at 3 months.

* H A M dash D: Follows a similar pattern to H A M dash A, with a peak at 2 months followed by a slight decrease.

* Columbia: No data bars are visible for this category.

* R F Q dash 8: Scores remain very low (under 5) across all time points with minimal fluctuation.

* Psychache: Starts high at T 0 (approx. 54), drops significantly until 1 month, spikes at 2 months (50), and drops again at 3 months.

* R M E T: Scores are relatively stable, fluctuating slightly between 25 and 30 across all time points.

* Q dash L E S dash Q: Shows a steady increase from T 0 to 1 month, a sharp drop at 2 months, and reaches its highest peak at 3 months (60).

* M A D R S: Starts at 25, decreases until 1 month, spikes at 2 months, and drops at 3 months.

Navigate back to Image 2:.

